# The Essential Role of the 3′ Terminal Template Base in the First Steps of Protein-Primed DNA Replication

**DOI:** 10.1371/journal.pone.0048257

**Published:** 2012-10-24

**Authors:** Irene Rodríguez, Elisa Longás, Miguel de Vega, Margarita Salas

**Affiliations:** Instituto de Biología Molecular “Eladio Viñuela” (Centro Superior de Investigaciones Científicas), Centro de Biología Molecular “Severo Ochoa” (Centro Superior de Investigaciones Científicas-Universidad Autónoma de Madrid), Cantoblanco, Madrid, Spain; University of Massachusetts Medical School, United States of America

## Abstract

Bacteriophages φ29 and Nf from *Bacillus subtilis* start replication of their linear genomes at both ends using a protein-primed mechanism by means of which the DNA polymerase initiates replication by adding dAMP to the terminal protein, this insertion being directed by the second and third 3′ terminal thymine of the template strand, respectively. In this work, we have obtained evidences about the role of the 3′ terminal base during the initiation steps of φ29 and Nf genome replication. The results indicate that the absence of the 3′ terminal base modifies the initiation position carried out by φ29 DNA polymerase in such a way that now the third position of the template, instead of the second one, guides the incorporation of the initiating nucleotide. In the case of Nf, although the lack of the 3′ terminal base has no effect on the initiation position, its absence impairs further elongation of the TP-dAMP initiation product. The results show the essential role of the 3′ terminal base in guaranteeing the correct positioning of replication origins at the polymerization active site to allow accurate initiation of replication and further elongation.

## Introduction

Some organisms such as bacteriophages and eukaryotic viruses contain replication origins formed by inverted terminal repeats (ITR) and a terminal protein (TP) covalently linked at both 5′ ends of their linear genomes [1], [2]. The location of the replication origins at the ends allows both strands to be replicated continuously from both ends in a symmetric fashion. For the initiation of replication, a free TP molecule forms a heterodimer with the DNA polymerase, providing the OH group of a specific Ser, Thr or Tyr residue to prime DNA replication from the very ends of the chromosome. After elongation, the TP remains covalently linked to the 5′ ends of the DNA, circumventing the problem of replication of linear chromosome ends [3]. The development of an *in vitro* replication system with purified proteins and TP-DNA from bacteriophage φ29 has been instrumental for the elucidation of this so-called protein-primed mechanism of DNA replication [1], [2].

The genome of φ29 is a linear dsDNA of 19285 bp, containing a TP of 31 kDa covalently linked to each 5′ end [4] that, together with a 6-bp ITR (3′-TTTCAT-5′) [5], [6] forms part of a minimal replication origin. Once the replication origins are specifically recognized by the heterodimer formed by the DNA polymerase and a free TP molecule [7], [8], the DNA polymerase catalyses the formation of a covalent bond between the initiating dAMP and the hydroxyl group of Ser^232^ of the TP, a reaction directed by the second T at the 3′ end of the template strand [9]. Then, to recover the template information corresponding to the terminal 3′ T, the TP-dAMP initiation product translocates backwards one position, the so-called sliding-back mechanism.

Bacteriophage Nf also belongs to the group of phages that infect *Bacillus* [10]. Its linear TP-DNA is 18754 bp long, containing a 6-bp ITR (3′ -TTTCAT-5′) [10], [11] and a 31 kDa TP at both 5′ ends [10]. In this case, the initiation of DNA replication occurs opposite the third 3′ terminal nucleotide of the template [12]. The recovery of the two terminal nucleotides mainly occurs by a step-wise sliding-back mechanism whereby the TP-dAMP formed opposite the third position is translocated one position back to pair with the second T, the third T directing again the formation of the TP-AA product. This product slides-back one position again, recovering the terminal 3′ T of the template. The sliding-back mechanism or variations of it, as the jumping-back that takes place in adenovirus [13] seems to have evolved in protein-priming replication systems as a general process to maintain full-length DNA [14], [2].

**Figure 1:**
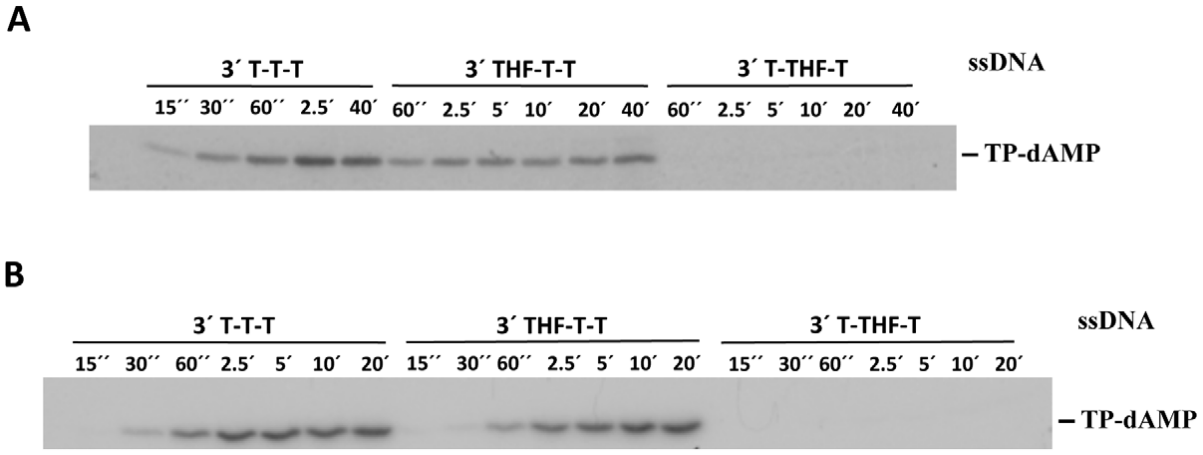
Formation of the TP-dAMP complex catalysed by either φ29 (A) or Nf (B) DNA polymerase. Using a single-stranded oligonucleotide with the chemically stable analogue of an abasic site, tetrahydrofuran (THF), at the 3′ end, in the presence of [α^32^P]dATP, both φ29 and Nf DNA polymerases were able to catalyze the formation of TP-dAMP product. These products were analysed by SDS-PAGE and autoradiography.

Phage φ29 DNA polymerase is the only protein-primed DNA-dependent replicase whose 3-D crystal structure has been solved, both as apoenzyme [15] and complexed to the TP [16]. The φ29 DNA polymerase/TP complex shows that TP has an extended structure complementary to the DNA polymerase surface. The TP is folded into a N-terminal domain, an intermediate domain that interacts with the DNA polymerase subdomain TPR1, and a C-terminal priming domain whose shape and electrostatic potential mimics DNA, occupying the DNA-binding cleft of the polymerase during initiation of DNA replication. This fact precludes the initiation at internal DNA sites, as an upstream 3′ template would sterically clash with the TP, restricting the beginning of DNA synthesis at the ends of the genome [16].

**Table 1:** Oligonucleotides used as template.

Oligonucleotide	Bacteriophague	Sequence 3′–5′
3′ T-T-T	*φ29*	TTTCATCCCATGTCGCTGTTG TATGTGGT
	*Nf*	TTTCATTCCAAGTTTCGTTTT AGCTGGGT
3′ THF-T-T	*φ29*	(THF)TTCATCCCATGTCGCTGTT GTATGTGGT
	*Nf*	(THF)TTCATTCCAAGTTTCGTTT TAGCTGGGT
3′ T-THF-T	*φ29*	T(THF)TCATCCCATGTCGCTGTT GTATGTGGT
	*Nf*	T(THF)TCATTCCAAGTTTCGTTT TAGCTGGGT
3′ THF-A-T	*φ29*	(THF)ATCATCCCATGTCGCTGTT GTATGTGGT
	*Nf*	(THF)ATCATTCCAAGTTTCGTTT TAGCTGGGT
3′ THF-T-A	*φ29*	(THF)TACATCCCATGTCGCTGTT GTATGTGGT
	*Nf*	(THF)TACATTCCAAGTTTCGTTT TAGCTGGGT

Single-stranded oligonucleotides containing the wild-type and modified sequences of the φ29 or Nf DNA right replication origin were obtained from Sigma. These oligonucleotides were used as template for both the initiation assay (TP-dNMP formation) and the truncated elongation assay. THF indicates the presence of tetrahydrofuran, which is a stable analogue of an abasic site.

All structural complexes of φ29 DNA polymerase with ssDNA show that the 3′ end of the ssDNA molecule reaches the DNA polymerase active site, while its 5′ terminus remains bound to the DNA polymerase in the tunnel that lies downstream of the active site in a non-sequence specific manner, suggesting that it alone cannot establish register of the template [17]. In the context of protein priming, where the polymerase adds the first nucleotide to residue Ser^232^ of the TP by pairing it with the second nucleotide from the 3′ end of the template [9], the priming domain of TP must position the template in the active site by sterically excluding it from the upstream duplex binding region of the polymerase, implying that the TP priming domain acts as a structural barrier. As a result of data obtained with chimerical TPs by swapping the priming domains of φ29 and Nf TPs [12], we know that TP and, specifically, its priming domain dictates the template position used to direct the initiation reaction. Thus, once the 3′ end of the template contacts the TP priming domain located at the duplex binding region, the corresponding internal T is placed at the catalytic site to direct formation of the TP-dAMP product.

To progress in the comprehension of this special mechanism to initiate replication, we have analysed here the influence of the 3′ terminal base of the template strand in the initiation and the first elongation steps of φ29 and Nf TP-DNA replication, by using highly purified DNA polymerase and TP of each phage, and template ssDNA oligonucleotides corresponding to either the wild-type sequence of the replication origins or variations of it.

**Figure 2:**
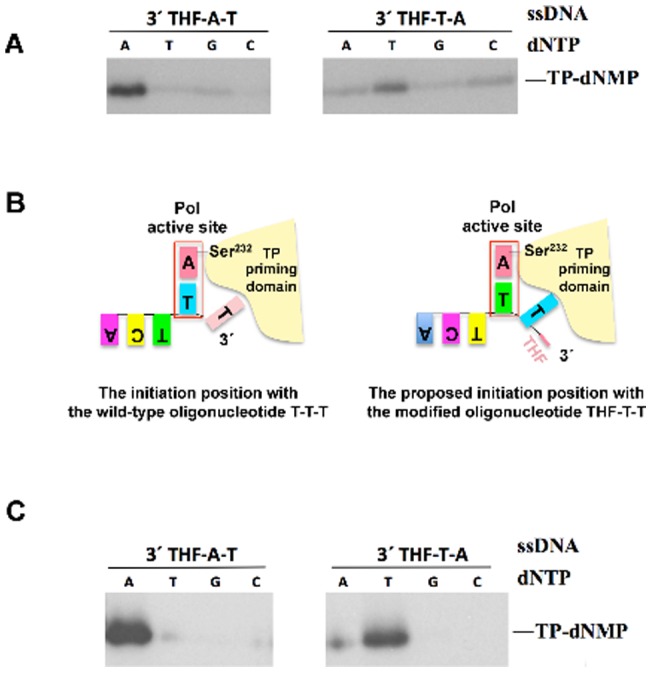
(A) The absence of the 3′ base at the template strand makes φ29 DNA polymerase to initiate opposite the third position. The different initiation products were detected by high resolution SDS-PAGE and analysed by autoradiography. *(* ***B*** *) Schematic representation of the placement of the 3′ end lacking the terminal base at the active site of* φ*29 DNA polymerase*. On the left side, the φ29 DNA polymerase catalyses the formation of the covalent bond between the initiating dAMP and the hydroxyl group of Ser^232^ of the TP, using as template the second T at the 3′ end of a wild-type template strand [9]. On the right side, the absence of the terminal base makes the template strand to enter the polymerization domain one position further, leading to the placement of the third 3′ nucleotide at the catalytic site to direct the formation of the initiation complex. (**C**) *The lack of the 3′ terminal base does not alter the initiation position by Nf DNA polymerase*. Bands corresponding to TP-dNMP products synthesized by Nf DNA polymerase were detected by high resolution SDS-PAGE.

## Materials and Methods

### Nucleotides and DNAs

Unlabelled nucleotides and dideoxynucleotides, [α-^32^P]dATP (3000 Ci/mmol), [α-^32^P]dTTP (3000 Ci/mmol), [α-^32^P]dGTP (3000 Ci/mmol) and [α-^32^P]dCTP (3000 Ci/mmol), were supplied by Amersham Biosciences. Single-stranded oligonucleotides containing the wild-type and modified sequences of the φ29 or Nf DNA right replication origin, used as template, were obtained from Sigma. Terminal protein-containing φ29 DNA (φ29 TP-DNA) was obtained as described [18].

**Figure 3:**
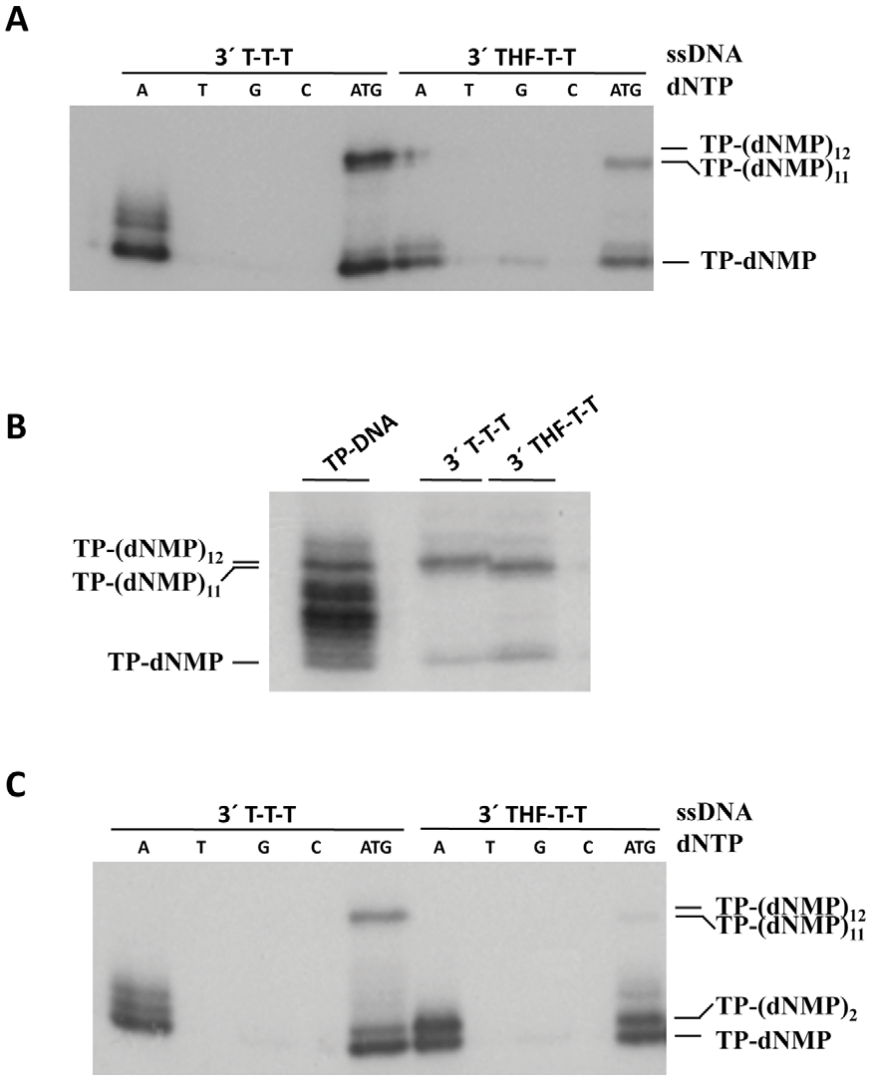
(A) The absence of the 3′ terminal base at the template oligonucleotide prevents the recovery of the 3′ terminal end by φ29 DNA polymerase. (**B**) Size of the products synthesized by φ29 DNA polymerase with the oligonucleotides 3′ T-T-T and 3′ THF-T-T. The length of the product synthesized by φ29 DNA polymerase with the oligonucleotides 3′ T-T-T and 3′ THF-T-T correspond with the intermediate replication products TP-(dNMP)_12_ and TP-(dNMP)_11_, respectively, as deduced from the comparison with the products obtained when the natural φ29 TP-DNA is used as template. (**C**) *The lack of the 3′ terminal base at the template strand blocks elongation of the initiation product by Nf DNA polymerase*. In all cases, the different elongation products were analysed by high resolution SDS-PAGE. The length at the different products is indicated.

### Proteins

Wild-type φ29 and Nf TPs were expressed in *E. coli* BL21(DE3) cells harboring the gene cloned into plasmid pT7-3 and further purified as described [19]. φ29 DNA polymerase mutant D12A/D66A and Nf DNA polymerase mutant D66A, both exonuclease deficient, were purified essentially as described [20].

### Template-Dependent *in Vitro* Formation of the Initiation Complex TP-dNMP

The incubation mixture contained, in 25 µl, 50 mM Tris-HCl (pH 7.5), 1 mM MnCl_2_, 20 mM ammonium sulphate, 4% (v/v) glycerol, 0.1 mg/ml BSA, 0.1 µM dNTP, 3 µCi of the corresponding labelled dNTP, 350 ng of the specified single-stranded oligonucleotide, 50 ng of φ29 or Nf TP and 50 ng of φ29 DNA polymerase mutant D12A/D66A or Nf DNA polymerase mutant D66A, and incubated for the indicated times at 30°C. The reactions were stopped by adding 10 mM EDTA and 0.1% SDS, filtered through Sephadex G-50 spin columns, and further analysed by SDS-PAGE as described [18]. The labelled bands corresponding to the TP-dNMP complex were detected by autoradiography.

### Truncated Elongation Assays

The incubation mixture contained, in 25 µl, 50 mM Tris-HCl (pH 7.5), 1 mM MnCl_2_, 20 mM ammonium sulphate, 4% (v/v) glycerol, 0.1 mg/ml BSA, 700 ng of the indicated single-stranded oligonucleotide or 500 ng of φ29 TP-DNA as template, 100 ng of purified TP and 100 ng of exonuclease-deficient φ29 or Nf DNA polymerase. In the case of the initiation reactions, 5 µM of the indicated dNTP and 4 µCi of the corresponding [α-^32^P]dNTP were used, and 5 µM dATP, dTTP and dGTP, 4 µCi of [α-^32^P]dNTP and 100 µM ddCTP in the case of the elongation reaction. In all cases, after incubation for five minutes at 30°C, reactions were stopped by adding EDTA to 10 mM. Samples were filtered through Sephadex G-50 spin columns in the presence of 0.1% SDS, and the excluded volume was analysed by 12% SDS-PAGE gel (360×280×0.5 mm) electrophoresis to obtain enough resolution to distinguish TP bound to the first elongation products. The length of the different products is indicated.

## Results and Discussion

### φ29 DNA polymerase changes the initiation position of replication in the absence of the 3′ terminal base

Previous studies showed that ssDNA containing replication origin sequences can support the template-directed TP-primed initiation reaction using DNA polymerase/TP heterodimers, having become a valuable tool to determine the precise initiation site, as well as to analyse the effect that nucleotide changes at the replication origins have in the first steps of DNA replication [9], [12], [13], [21], [22], [23]. This fact has led us to study the influence of the base of the 3′ terminal nucleotide in the template-directed formation of TP-dAMP (initiation complex). For this, we used a single-stranded oligonucleotide in which the 3′-terminal T was replaced by the chemically stable analogue of an abasic site, tetrahydrofuran (THF; THF-T-T oligonucleotide) (see **Table** **I**). To prevent degradation of the template oligonucleotides, exonuclease deficient DNA polymerases, φ29 D12A/D66A [24] and Nf D66A [25], were used. In the presence of [α-^32^P]dATP, both φ29 (**Figure** **1A**) and Nf (**Figure** **1B**) DNA polymerases were able to catalyse the formation of TP-dAMP in the absence of the 3′ terminal base of the template, although in the case of φ29 DNA polymerase in a lower extent than with the unmodified oligonucleotide (**Figure** **1A**). As expected, the absence of the base at the penultimate 3′ nucleotide of the template (T-THF-T oligonucleotide) precluded the initiation reaction by the φ29 DNA polymerase/TP heterodimer, as this enzyme has been shown to initiate opposite the second 3′ terminal position of the template [9]. Interestingly, the presence of an abasic site in the penultimate position impaired initiation by Nf DNA polymerase, despite the fact that formation of the TP-dAMP product by this polymerase is guided by the third position of the template (**Figure** **1B**). This fact could suggest that proper stability/orientation of the third T at the Nf DNA polymerase depends on the presence of the 3′ neighbour base.

Having established that φ29 DNA polymerase is able to initiate using as template the THF-T-T oligonucleotide, the next step was to determine which of the 3′ terminal Ts directed the initiation reaction. For this, single changes into A at the second or third position of the template were introduced and assayed for TP-primed initiation with each of the four [α-^32^P]dNTPs. Unexpectedly, the substitution of the second T (THF-A-T oligonucleotide) made the polymerase to preferentially catalyse the formation of TP-dAMP product (**Figure** **2A**), suggesting that the polymerase was using the third T as template of the initiation reaction instead of the second one. To confirm this possibility, the same kind of experiment was carried out changing the third T on the template into A. As it can be observed in **Figure** **2A**, now the φ29 DNA polymerase catalysed preferentially the formation of the TP-dTMP product. These results led us to propose a model in which the absence of the terminal base makes the template strand to enter the polymerization domain one position further, making the third 3′ nucleotide to be allocated at the catalytic site to direct the formation of the initiation complex (see **Figure** **2B**). As shown in **Figure** **2A**, the fidelity of φ29 DNA polymerase on the THF-T-A oligonucleotide was lower than that obtained with the oligonucleotide THT-A-T. The change of the third T into A (THF-T-A oligonucleotide) gave rise, in addition to the expected initiation product TP-dTMP, to the formation of smaller amounts of TP-dAMP, TP-dGMP and TP-dCMP initiation products. This result could indicate that the stability of the templating base is influenced by the sequence context in such a way that the position previous to the templating base could affect the incorporation of the initiating nucleotide. In this sense, it is possible that the presence of a pyrimidine previous to the initiation position in oligonucleotide THF-T-A decreases the fidelity of φ29 DNA polymerase by reducing the proper stabilization of the templating nucleotide that directs the initiation position.

Intriguingly, in spite of the high percentage of sequence similarity between φ29 and Nf DNA polymerase/TP heterodimers [26], the latter shows a behaviour quite different from that of the φ29 one with respect to the absence of the 3′ terminal base of the template. Nf DNA polymerase does not seem to require the terminal base for the proper localization of the template oligonucleotide since, in the absence of the terminal base, Nf DNA polymerase is still able to use the 3′ third position as template nucleotide, being TP-dAMP and TP-dTMP the main initiation products obtained with THF-A-T and THF-T-A oligonucleotides, respectively (**Figure** **2C**). This last result indicates that the presence of THF at the 3′ end does not modify the initiation position by Nf DNA polymerase.

### Nf DNA polymerase is not able to elongate the initiation product on oligonucleotides with an abasic site at the 3′ terminus

It has been reported that the TP-dAMP initiation product formed using the second and third terminal nucleotide by the φ29 and Nf systems, respectively, is not directly elongated from the initiation site [9], [25]. In φ29, the sliding-back mechanism has evolved to recover the terminal T of the template through translocation of the initiation complex one position back, enabling to use again as template the penultimate T to direct the insertion of the second nucleotide [9]. In Nf, the related step-wise sliding-back mechanism allows the recovery of the first two nucleotides by means of two consecutive sliding-back steps [25]. These processes have to take place before definitive elongation of the initiation products occurs.

Once established that the absence of the 3′ terminal base makes the φ29 DNA polymerase to initiate opposite the third nucleotide, it was necessary to find out whether the first two nucleotides were recovered during the first steps of replication. To address this question, elongation of the initiation product TP-dAMP was studied in the presence of dATP, dTTP, dGTP and ddCTP, using oligonucleotides containing either the wild-type φ29 and Nf replication origins or the variants bearing a 3′ terminal THF. Thus, if the 2 nucleotides are recovered before elongation, the maximum length of the replication product in the presence of ddCTP obtained with φ29 DNA polymerase would be TP-(dNMP)_12_ with the oligonucleotide 3′ T-T-T, as previously described [9]. Firstly, to analyse that this elongation is the result of the synthesis onto the TP-dAMP product, each nucleotide was provided separately. As shown in **Figure** **3A**, the φ29 DNA polymerase gave rise to initiation products only in the presence of dATP, TP-dAMP being the main product, although small amounts of TP(dAMP)_3_-TP(dNMP)_4_ complexes were also detectable, the latter as a result of misincorporation in front of the fourth position due to the use of the 3′-5′ exonuclease deficient φ29 DNA polymerase mutant D12A/D66A [24]. These results indicate that the longer elongation products that could be yielded in the presence of the four nucleotides would be the result of the direct elongation of TP-dAMP initiation complex (see below). On the other hand, the lack of initiation products when each of the other three [α^32^P]dNTPs are used indicates that the absence of the 3′ terminal base does not affect the fidelity of the initiation reaction. As seen in **Figure** **3A and B**, with the wild-type φ29 sequence, and in the presence of dATP, dTTP, dGTP and ddCTP, replication was truncated at TP(dAMP)_12_. The use of the natural φ29 TP-DNA as template rendered bands corresponding to intermediate products and allowed us the assignment of the elongation band (**Figure** **3B**). On the contrary, the maximum length of the replication product obtained with oligonucleotide THF-T-T was TP-(dNMP)_11_ (**Figure** **3A and B**), suggesting that once the TP-dAMP is formed opposite the third position of the template, the initiation complex slides-back one position, recovering the penultimate T, and is then elongated. The lack of a second sliding-back step to recover the 3′ terminal position could be due either to the incapacity to hybridise the TP-(dAMP)_2_ product opposite the 3′ THF-T (see **Figure** **2B**), which would require the breakage of the hydrogen-bond of two base pairs, a situation energetically unfavoured, or because the polymerase recognizes the TP-(dAMP)_2_ bound to the second and third positions as the functional one, due to the lack of a 3′ terminal base. Therefore, it seems that the THF at the 3′ end does not preclude the sliding-back movement of the TP-dAMP product opposite the penultimate position of the template but does not allow the sliding-back opposite the THF.

In the case of the Nf DNA polymerase, when the initiation with the different nucleotides on the T-T-T oligonucleotide was assayed, bands corresponding to TP-(dAMP)_1–3_ were observed only in the presence of [α-^32^P]dATP. As it occurred with φ29 DNA polymerase, TP-(dNMP)_12_ was the longest replication product observed in the presence of dATP, dTTP, dGTP and ddCTP, indicating the recovery of the two terminal nucleotides (**Figure** **3C**). Interestingly, when Nf DNA polymerase had to elongate the THF-T-T oligonucleotide, in contrast to φ29 DNA polymerase, replication was blocked mainly at position 2. This result indicates that the TP-dAMP initiation complex slided-back once, but the absence of a 3′ terminal base precluded the second sliding-back step. In addition, instead of elongating directly the TP-(dAMP)_2_ product, as the Nf DNA polymerase does when it cannot perform the second sliding-back step [12], the absence of the 3′ terminal base hampered elongation. This result could be pointing to either a misalignment of the template at the catalytic site, or to the requirement of a terminal base to allow the DNA polymerase to proceed further.

The present study leads us to propose an essential role for the base of the 3′ terminal nucleotide to allow initiation at the corresponding penultimate nucleotide in the case of φ29, and to allow elongation in the case of Nf during the first steps of the protein-primed replication process. In addition, the presence of a base previous to the initiation site in Nf seems to be required to allow proper placement of the templating base for initiation.

## References

[1] Salas M (1991) Protein-priming of DNA replication. Annu Rev Biochem 60, 39–71. 10.1146/annurev.bi.60.070191.000351 1883199

[2] Salas M, Miller J, Leis J, DePamphilis M (1996) Mechanisms for priming DNA synthesis. Cold Spring Harbor Laboratory Press, New York.

[3] Kornberg A, Baker T (1992) DNA replication. 2nd edition. Freeman (San Francisco).

[4] Salas M, Mellado RP, Viñuela E, Sogo JM (1978) Characterization of a protein covalently linked to the 5′ termini of the DNA of *Bacillus subtilis* phage φ29. J Mol Biol 119, 269–291. 10.1016/0022-2836(78)90438-2 416224

[5] Escarmís C, Salas M (1982) Nucleotide sequence of the early genes 3 and 4 of bacteriophage φ29. Nucleic Acids Res 10, 5785–5798. 10.1093/nar/10.19.5785 PMC320930 6292852

[6] Yoshikawa H, Ito J (1982) Nucleotide sequence of the major early region of bacteriophage φ29. Gene 17, 323–335. 10.1016/0378-1119(82)90149-4 6809534

[7] Blanco L, Prieto I, Gutiérrez J, Bernad A, Lázaro JM et al. (1987) Effect of NH_4_ ^+^ ions on φ29 DNA-protein p3 replication: Formation of a complex between the terminal protein and the DNApolymerase. J Virol 61, 3983–3991. 10.1128/jvi.61.12.3983-3991.1987 PMC256019 3682063

[8] Gutiérrez J, Vinos J, Prieto I, Méndez E, Hermoso JM et al.. (1986) Signals in the φ29 DNA-terminal protein template for the initiation of phage φ29 DNA replication. Virol 155, 474–483. 10.1016/0042-6822(86)90209-6 3097958

[9] Méndez J, Blanco L, Esteban JA, Bernad A, Salas M (1992) Initiation of φ29 DNA replication occurs at the second 3′ nucleotide of the linear template: a sliding-back mechanism for protein-primed DNA replication. Proc Natl Acad Sci U S A 89, 9579–9583. 10.1073/pnas.89.20.9579 PMC50175 1409668

[10] Yoshikawa H, Ito J (1981) Terminal proteins and short inverted terminal repeats of the small *Bacillus* bacteriophage genomes. Proc Natl Acad Sci USA 78, 2596–2600. 10.1073/pnas.78.4.2596 PMC319396 6941313

[11] Yoshikawa H, Garvey KJ, Ito J (1985) Nucleotide sequence analysis of DNA replication origins of the small *Bacillus* bacteriophages: Evolutionary relationships. Gene 37, 125–130. 10.1016/0378-1119(85)90264-1 3932129

[12] Longás E, Villar L, Lázaro JM, de Vega M, Salas M (2008) Phage φ29 and Nf terminal protein-priming domain specifies the internal template nucleotide to initiate DNA replication. Proc Natl Acad Sci USA 47, 18290–18295. 10.1073/pnas.0809882105 PMC2587616 19011105

[13] King AJ, van der Vliet PC (1994) A precursor terminal protein-trinucleotide intermediate during initiation of adenovirus DNA replication: Regeneration of molecular ends in vitro by a jumping back mechanism. EMBO J 13, 5786–5792. 10.1002/j.1460-2075.1994.tb06917.x PMC395545 7988575

[14] Salas M (1999) Mechanisms of initiation of linear DNA replication in prokaryotes. Genet Eng (NY) 21, 159–171. 10.1007/978-1-4615-4707-5_8 10822496

[15] Kamtekar S, Berman AJ, Wang J, Lázaro JM, de Vega M et al.. (2004) Insights into strand displacement and processivity from the crystal structure of the protein-primed DNA polymerase of bacteriophage φ29. Mol Cell 16, 609–618. 10.1016/j.molcel.2004.10.019 15546620

[16] Kamtekar S, Berman AJ, Wang J, Lázaro JM, de Vega M et al.. (2006) The φ29 DNA polymerase:protein-primer structure suggests a model for the initiation to elongation transition. EMBO J 25, 1335–1343. 10.1038/sj.emboj.7601027 PMC1422159 16511564

[17] Berman AJ, Kamtekar S, Goodman JL, Lázaro JM, de Vega M et al.. (2007) Structures of φ29 DNA polymerase complexed with substrate: the mechanism of translocation in B-family polymerases. EMBO J. 26, 3494–3505. 10.1038/sj.emboj.7601780 PMC1933411 17611604

[18] Peñalva MA, Salas M (1982) Initiation of phage φ29 DNA replication in vitro: formation of a covalent complex between the terminal protein, p3, and 5′-dAMP. Proc Natl Acad Sci USA 79, 5522–5526. 10.1073/pnas.79.18.5522 PMC346936 6813861

[19] Mencía M, Gella P, Camacho A, de Vega M, Salas M (2011) Terminal protein-primed amplification of heterologous DNA with a minimal replication system based on phage phi29. Proc Natl Acad Sci USA 46, 18655–18660. 10.1073/pnas.1114397108 PMC3219123 22065756

[20] Lázaro JM, Blanco L, Salas M (1995) Purification of bacteriophage φ29 DNA polymerase. Methods Enzymol 262, 42–49. 10.1016/0076-6879(95)62007-9 8594366

[21] Illana B, Zaballos A, Blanco L, Salas M (1998) The The RGD sequence in phage ø29 terminal protein is required for interaction with ø29 DNA polymerase. Virol 248(1), 12–19. 10.1006/viro.1998.9276 9705251

[22] Martín AC, Blanco L, García P, Salas M, Méndez J (1996) In vitro protein-primed initiation of pneumococcal phage Cp-1 DNA replication occurs at the third 3′ nucleotide of the linear template: A stepwise sliding-back mechanism. J Mol Biol 260, 369–377. 10.1006/jmbi.1996.0407 8757800

[23] Caldentey J, Blanco L, Bamford DH, Salas M (1993) In vitro replication of bacteriophage PRD1 DNA. Characterization of the protein-primed initiation site. Nucleic Acids Res 21, 3725–3730. 10.1093/nar/21.16.3725 PMC309875 8367287

[24] Bernad A, Blanco L, Lázaro JM, Martín G, Salas M (1989) A conserved 3′-5′ exonuclease active site in prokaryotic and eukaryotic DNA polymerases. Cell 59, 219–228. 10.1016/0092-8674(89)90883-0 2790959

[25] Longás E, de Vega M, Lázaro JM, Salas M (2006) Functional characterization of highly processive protein-primed DNA polymerases from phages Nf and GA-1, endowed with a potent strand displacement capacity. Nucleic Acids Res 34, 6051–6063. 10.1093/nar/gkl769 PMC1635332 17071961

[26] González-Huici V, Salas M, Hermoso JM (2000) Sequence requirements for protein primed initiation and elongation of phage φ29 DNA replication. J Biol Chem 275, 40547–40553. 10.1074/jbc.M007170200 11006291

